# Supplementary material for: Diversity and inclusion for the All of Us research program: A scoping review
Source: PLoS One. 2020 Jul 1;15(7):e0234962. doi: 10.1371/journal.pone.0234962 (PMC7329113; doi:10.1371/journal.pone.0234962)
Supplement: S1 Table — (DOCX) [file pone.0234962.s001.docx]

**S1 Table.** **Search Strategies by Diversity Category and January 17, 2020 Retrieval Counts**

| **Search Category** | **Search Strategy** | **1/17/2020 through 1/21/2020 Retrieval Counts** |
| --- | --- | --- |
| **Access to Care** | ("healthcare disparities"[MAJR] OR "Health care quality, access, and evaluation"[MAJR] OR "Health services accessibility"[MAJR] OR "medical indigency"[MAJR] OR "medically uninsured"[MAJR] OR "access to care"[tiab] OR "health care access"[tiab] OR "healthcare access"[tiab] OR "health care accessibility"[tiab] OR "uninsured"[ti] OR "insured"[ti] OR medicaid[ti]) | 147834 |
|  | ("healthcare disparities"[mesh] OR "Health care quality, access, and evaluation"[mesh] OR "Health services accessibility"[mesh] OR "medical indigency"[mesh] OR "medically uninsured"[mesh] OR "access to care"[tiab] OR "health care access"[tiab] OR "healthcare access"[tiab] OR "health care accessibility"[tiab]) AND ("participation"[ti] OR representation[ti] OR under-representation[tiab] OR represented[ti] OR underrepresentation[tiab] OR underrepresented[tiab] OR under-represented[tiab] OR enrollment[ti] OR under-enrollment[tiab] OR representation[tiab] OR diversity[ti] OR inclusion[ti] OR exclusion[ti]) AND (research[tiab] OR clinical[tiab] OR study[tiab] OR studies[tiab] OR trial[tiab] OR trials[tiab]) AND (review[tiab] OR review[ptyp] OR “systematic review”[tiab] OR meta-analysis[tiab]) | 3662 |
|  | ("healthcare disparities"[MAJR] OR "Health care quality, access, and evaluation"[MAJR] OR "Health services accessibility"[MAJR] OR "medical indigency"[MAJR] OR "medically uninsured"[MAJR] OR "access to care"[tiab] OR "health care access"[tiab] OR "healthcare access"[tiab] OR "health care accessibility"[tiab]) AND ("participation"[ti] OR representation[ti] OR under-representation[tiab] OR represented[ti] OR underrepresentation[tiab] OR underrepresented[tiab] OR under-represented[tiab] OR enrollment[ti] OR under-enrollment[tiab] OR representation[tiab] OR diversity[ti] OR inclusion[ti] OR exclusion[ti]) AND (research[tiab] OR clinical[tiab] OR study[tiab] OR studies[tiab] OR trial[tiab] OR trials[tiab]) AND (review[tiab] OR review[ptyp] OR “systematic review”[tiab] OR meta-analysis[tiab]) | 1683 |
|  | ("access to care"[tiab] OR "health care access"[tiab] OR "healthcare access"[tiab] OR "health care accessibility"[tiab] OR "uninsured"[ti] OR "insured"[ti] OR medicaid[ti]) AND ("participation"[ti] OR representation[ti] OR under-representation[tiab] OR represented[ti] OR underrepresentation[tiab] OR underrepresented[tiab] OR under-represented[tiab] OR enrollment[ti] OR under-enrollment[tiab] OR representation[tiab] OR diversity[ti] OR inclusion[ti] OR exclusion[ti]) AND (research[tiab] OR clinical[tiab] OR study[tiab] OR studies[tiab] OR trial[tiab] OR trials[tiab]) | 274 |
|  | (insurance[ti]) AND ("participation"[ti] OR representation[ti] OR under-representation[tiab] OR represented[ti] OR underrepresentation[tiab] OR underrepresented[tiab] OR under-represented[tiab] OR enrollment[ti] OR under-enrollment[tiab] OR representation[tiab] OR diversity[ti] OR inclusion[ti] OR exclusion[ti]) AND (research[tiab] OR clinical[tiab] OR study[tiab] OR studies[tiab] OR trial[tiab] OR trials[tiab]) | 123 |
| **Age** | ((age[ti] OR children[ti] OR adolescent[ti] OR adolescents[ti] OR teens[ti] OR teenager[ti] OR “young adults”[ti] OR youth[ti] OR teenagers[ti] OR elderly[ti] OR “older adults”[ti] OR geriatric[ti] OR geriatrics[ti]) AND ("exclusion"[ti] OR "inclusion"[ti] OR "participation in research"[tiab] OR "recruit"[ti] OR "recruited"[tiab] OR "recruiting"[tiab] OR "recruitment"[tiab] OR "research participant"[tiab] OR "research participants"[tiab] OR "research participation"[tiab] OR "research subject"[tiab] OR "research subjects"[tiab] OR "underenroll"[tiab] OR "underenrolled"[tiab] OR "underrepresented"[tiab] OR under-representation[tiab] OR underrepresentation[tiab] OR under-enrollment[tiab]) AND ( "2012/01/01"[PDat] : "2025/01/01"[PDat] ) AND English[la] AND (review[ptyp] OR systematic[tiab] OR review[tiab])) NOT Animals[Mesh:noexp] | 950 |
| **Annual Household Income** | ("indigency"[tiab] OR "indigent"[tiab] OR "indigents"[tiab]OR "income"[mesh] OR "income distribution"[tiab] OR "low income population"[tiab] OR "low-income population"[tiab] OR "medical indigency"[mesh] OR "poverty"[mesh] OR "poverty level"[tiab] OR "uncompensated care"[mesh] OR "indigent care"[tiab] OR “vulnerable populations”[mesh] OR “poverty areas”[tiab] OR “low income”[tiab] OR “low-income”[tiab]) AND ("Research participation"[tiab] OR "participation in research"[tiab] OR "underenrolled"[tiab] OR "research participant"[tiab] OR "patient selection"[mesh] OR "underenroll"[tiab] OR "recruit"[tiab] OR "research subjects"[mesh]) | 1614 |
|  | ("indigency"[tiab] OR "indigent"[tiab] OR "indigents"[tiab]OR "income"[mesh] OR "income distribution"[tiab] OR "low income population"[tiab] OR "low-income population"[tiab] OR "medical indigency"[mesh] OR "poverty"[mesh] OR "poverty level"[tiab] OR "uncompensated care"[mesh] OR "indigent care"[tiab] OR “vulnerable populations”[mesh] OR “poverty areas”[tiab] OR “low income”[tiab] OR “low-income”[tiab]) AND ("exclusion"[tiab] OR "inclusion"[tiab] OR "human subjects"[tiab] OR "participation in research"[tiab] OR "patient selection"[mesh] OR "recruit"[tiab] OR "recruited"[tiab] OR "recruiting"[tiab] OR "recruitment"[tiab] OR "research participant"[tiab] OR "research participants"[tiab] OR "research participation"[tiab] OR "research subjects"[mesh] OR "research subject"[tiab] OR "research subjects"[tiab] OR "underenroll"[tiab] OR "underenrolled"[tiab] OR "underrepresented"[tiab] OR under-representation[tiab]) | 8372 |
| **Disability** | ("Child Development Disorders, Pervasive" [mesh] OR "Community Mental Health Services" [mesh] OR "Developmental Disabilities" [mesh] OR "Disabled Children" [mesh] OR "Disabled Persons" [mesh] OR "Health Services for Persons with Disabilities" [mesh] OR "Intellectual Disability" [mesh] OR "Learning Disorders" [mesh] OR "Mentally Disabled Persons" [mesh] OR "Mental Disorders" [mesh] OR "Mentally Ill Persons" [mesh] OR "Persons with Hearing Impairments" [mesh] OR "Visually Impaired Persons" [mesh] OR "Cognitive Dysfunction" [Mesh] OR disabled[tiab] OR disability[tiab] OR disabilities[tiab] OR "cognitive impairment"[tiab] OR "cognitively impaired"[tiab] OR handicapped[tiab] OR "Cognitive Dysfunction" [tiab] OR "learning disorder"[tiab] OR "learning disorders"[tiab] OR "mentally ill"[tiab] OR "hearing impairment"[tiab] OR "hearing impairments"[tiab] OR "visually impaired"[tiab] OR "visual impairments"[tiab]) AND ("participation"[ti] OR representation[ti] OR under-representation[tiab] OR represented[ti] OR underrepresentation[tiab] OR underrepresented[tiab] OR under-represented[tiab] OR enrollment[ti] OR under-enrollment[tiab] OR representation[tiab] OR diversity[ti] OR inclusion[ti] OR exclusion[ti]) AND (research[tiab] OR clinical[tiab] OR study[tiab] OR studies[tiab] OR trial[tiab] OR trials[tiab]) AND (review[tiab] OR review[ptyp] OR “systematic review”[tiab] OR meta-analysis[tiab]) | 1157 |
|  | (disabled[ti] OR disability[ti] OR disabilities[ti] OR "cognitive impairment"[ti] OR "cognitively impaired"[ti] OR handicapped[ti] OR "Cognitive Dysfunction" [ti] OR "learning disorder"[ti] OR "learning disorders"[ti] OR "mentally ill"[ti] OR "hearing impairment"[ti] OR "hearing impairments"[ti] OR "visually impaired"[ti] OR "visual impairments"[ti] OR Dementia[ti]) AND ("participation"[ti] OR representation[ti] OR under-representation[tiab] OR represented[ti] OR underrepresentation[tiab] OR underrepresented[tiab] OR under-represented[tiab] OR enrollment[ti] OR under-enrollment[tiab] OR representation[tiab] OR diversity[ti] OR inclusion[ti] OR exclusion[ti]) AND (research[tiab] OR clinical[tiab] OR study[tiab] OR studies[tiab] OR trial[tiab] OR trials[tiab]) | 1052 |
|  | (disabled[ti] OR disability[ti] OR disabilities[ti] OR "cognitive impairment"[ti] OR "cognitively impaired"[ti] OR handicapped[ti] OR "Cognitive Dysfunction" [ti] OR "learning disorder"[ti] OR "learning disorders"[ti] OR "mentally ill"[ti] OR "hearing impairment"[ti] OR "hearing impairments"[ti] OR "visually impaired"[ti] OR "visual impairments"[ti] OR Dementia[ti]) AND (representation[ti] OR under-representation[tiab] OR represented[ti] OR underrepresentation[tiab] OR underrepresented[tiab] OR under-represented[tiab] OR enrollment[ti] OR under-enrollment[tiab] OR representation[tiab] OR diversity[ti] OR inclusion[ti] OR exclusion[ti]) AND (research[tiab] OR clinical[tiab] OR study[tiab] OR studies[tiab] OR trial[tiab] OR trials[tiab]) | 572 |
|  | (disabled[ti] OR disability[ti] OR disabilities[ti] OR "cognitive impairment"[ti] OR "cognitively impaired"[ti] OR handicapped[ti] OR "Cognitive Dysfunction" [ti] OR "learning disorder"[ti] OR "learning disorders"[ti] OR "mentally ill"[ti] OR "hearing impairment"[ti] OR "hearing impairments"[ti] OR "visually impaired"[ti] OR "visual impairments"[ti]) AND ("participation"[ti] OR representation[ti] OR under-representation[tiab] OR represented[ti] OR underrepresentation[tiab] OR underrepresented[tiab] OR under-represented[tiab] OR enrollment[ti] OR under-enrollment[tiab] OR representation[tiab] OR diversity[ti] OR inclusion[ti] OR exclusion[ti] OR dementia[ti]) AND (research[tiab] OR clinical[tiab] OR study[tiab] OR studies[tiab] OR trial[tiab] OR trials[tiab]) AND (review[tiab] OR review[ptyp] OR “systematic review”[tiab] OR meta-analysis[tiab]) | 544 |
| **Educational Attainment** | ("GED equivalent"[ti] OR "High school equivalency diploma"[ti] OR "high school graduate"[ti] OR "high school graduates"[ti] OR "Never completed high school"[ti] OR "Not a high school graduate"[ti] OR "non-high school graduate"[ti] OR "non-high school graduates"[ti] OR "School age population"[ti] OR "School-age population"[ti] OR "Academic status"[ti] OR "Education level"[ti] OR "Educational attainment"[ti] OR "Educational differences"[ti] OR "Educational Levels"[ti] OR "Educational status"[ti] OR "general equivalency development"[ti] OR "general equivalency diploma"[ti] OR "health literacy"[ti] OR "High school education"[ti] OR "Less educated"[ti] OR "Less well-educated"[ti] OR "Level of education"[ti] OR "Literacy"[mesh] OR "Literacy"[ti] OR "Low education"[ti] OR "Lower education"[ti] OR "Numeracy"[ti] OR "Primary schooling"[ti] OR "Secondary education"[ti] OR "student drop-out"[ti] OR "student dropouts"[mesh] OR GED[ti] OR "lower educational"[ti] OR "lack of education"[ti] OR "elementary education"[ti]) | 11155 |
|  | “educational attainment”[ti] | 762 |
|  | ("GED equivalent"[tiab] OR "High school equivalency diploma"[tiab] OR "high school graduate"[tiab] OR "high school graduates"[tiab] OR "Never completed high school"[tiab] OR "Not a high school graduate"[tiab] OR "non-high school graduate"[tiab] OR "non-high school graduates"[tiab] OR "School age population"[tiab] OR "School-age population"[tiab] OR "Academic status"[tiab] OR "Education level"[tiab] OR "Educational attainment"[tiab] OR "Educational differences"[tiab] OR "Educational Levels"[tiab] OR "Educational status"[tiab] OR "general equivalency development"[tiab] OR "general equivalency diploma"[tiab] OR "health literacy"[tiab] OR "High school education"[tiab] OR "Less educated"[tiab] OR "Less well-educated"[tiab] OR "Level of education"[tiab] OR "Literacy"[mesh] OR "Literacy"[tiab] OR "Low education"[tiab] OR "Lower education"[tiab] OR "Numeracy"[tiab] OR "Primary schooling"[tiab] OR "Schooling"[tiab] OR "Secondary education"[tiab] OR "student drop-out"[tiab] OR "student dropouts"[mesh] OR GED[tiab] OR "lower educational"[tiab] OR "lack of education"[tiab] OR "elementary education"[tiab]) AND ("exclusion"[tiab] OR "inclusion"[tiab] OR "human subjects"[tiab] OR "participation in research"[tiab] OR "patient selection"[mesh] OR "recruit"[tiab] OR "recruited"[tiab] OR "recruiting"[tiab] OR "recruitment"[tiab] OR "research participant"[tiab] OR "research participants"[tiab] OR "research participation"[tiab] OR "research subjects"[mesh] OR "research subject"[tiab] OR "research subjects"[tiab] OR "underenroll"[tiab] OR "underenrolled"[tiab] OR "underrepresented"[tiab] OR under-representation[tiab]) AND (review[tiab] OR review[ptyp] OR “systematic review”[tiab] OR meta-analysis[tiab]) | 807 |
| Gender Identity | ("Gender Dysphoria"[Mesh] OR "gender dysphoria"[tiab] OR "Gender Identity"[Mesh] OR "gender identity"[tiab] OR "Health Services for Transgender Persons"[Mesh] OR "transgender"[tiab] OR "Sexual and Gender Disorders"[Mesh] OR "Transgender Persons"[Mesh] OR "Transsexualism"[Mesh] OR "genderqueer"[tiab] OR "non-binary"[tiab] OR "gender non-conforming"[tiab] OR "trans male"[tiab] OR "trans man"[tiab] OR "trans female"[tiab] OR "trans woman"[tiab]) AND ("exclusion"[tiab] OR "inclusion"[tiab] OR "human subjects"[tiab] OR "participation in research"[tiab] OR "patient selection"[mesh] OR "recruit"[tiab] OR "recruited"[tiab] OR "recruiting"[tiab] OR "recruitment"[tiab] OR "research participant"[tiab] OR "research participants"[tiab] OR "research participation"[tiab] OR "research subjects"[mesh] OR "research subject"[tiab] OR "research subjects"[tiab] OR "underenroll"[tiab] OR "underenrolled"[tiab] OR "underrepresented"[tiab] OR under-representation[tiab] OR underrepresentation[tiab] OR under-enrollment[tiab] OR representation[tiab]) | 1773 |
| Geography | ("Hospitals, Rural"[mesh] OR "medically underserved area"[mesh] OR "poverty areas"[mesh] OR "rural health"[mesh] OR "rural health services"[mesh] OR "rural nursing"[mesh] OR "rural population"[mesh] OR "rural"[tiab] OR "urban"[tiab] OR "medically underserved area"[tiab]) AND ("exclusion"[tiab] OR "inclusion"[tiab] OR "human subjects"[tiab] OR "participation in research"[tiab] OR "patient selection"[mesh] OR "recruit"[tiab] OR "recruited"[tiab] OR "recruiting"[tiab] OR "recruitment"[tiab] OR "research participant"[tiab] OR "research participants"[tiab] OR "research participation"[tiab] OR "research subjects"[mesh] OR "research subject"[tiab] OR "research subjects"[tiab] OR "underenroll"[tiab] OR "underenrolled"[tiab] OR "underrepresented"[tiab] OR under-representation[tiab]) | 15666 |
|  | ("participation"[ti] OR representation[ti] OR under-representation[tiab] OR represented[ti] OR underrepresentation[tiab] OR underrepresented[tiab] OR under-represented[tiab] OR enrollment[ti] OR under-enrollment[tiab] OR representation[tiab] OR diversity[ti] OR inclusion[ti] OR exclusion[ti]) AND (research[tiab] OR clinical[tiab] OR study[tiab] OR studies[tiab] OR trial[tiab] OR trials[tiab]) AND ("Hospitals, Rural"[mesh] OR "medically underserved area"[mesh] OR "poverty areas"[mesh] OR "rural health"[mesh] OR "rural health services"[mesh] OR "rural nursing"[mesh] OR "rural population"[mesh] OR "rural"[tiab] OR "urban"[tiab] OR "medically underserved area"[tiab]) | 2266 |
| Race/Ethnicity | (("Cultural Diversity"[MeSH] OR "Ethnic Groups"[MeSH] OR "Minority Groups"[MeSH] OR "Minority Health"[MeSH] OR “Cultural diversity”[tiab] OR “Racial and ethnic minorities”[tiab] OR “Ethnic minorities”[tiab] OR “Racial minority”[tiab] OR “Racial differences”[tiab] OR “Racial differences”[tiab] OR “Racial”[tiab] OR “Ethnic minority”[tiab] OR “Ethnically diverse”[tiab] OR “Minorities”[tiab] OR “Underserved”[tiab] OR “Minority”[tiab] OR “Racially diverse”[tiab] OR “Diverse population”[tiab] OR "African Americans"[mesh] OR "african continental ancestry group"[mesh] OR “blacks”[tiab] OR “black”[tiab] OR “African-American”[tiab] OR "African Americans”[tiab] OR “ negro”[tiab] OR “negroes”[tiab] OR "negroid race”[tiab] OR “African ancestry”[tiab] OR “Hispanic Americans”[mesh] OR “Mexican Americans”[mesh] OR “Hispanic”[tiab] OR “Latino”[tiab] OR “Hispanics”[tiab] OR “Latinas”[tiab] OR “Latinos”[tiab] OR “Latina”[tiab] OR “Latino”[tiab] OR “Latin American”[tiab] OR “Mexican Americans”[tiab] OR ”Mexican American”[tiab] OR “chicanos”[tiab] OR “chicanas”[tiab] OR “chicano”[tiab] OR “chicana”[tiab] OR “Cuban descent”[tiab] OR “Puerto Rican descent”[tiab] OR “Mexican descent”[tiab] OR “Hispanic Puerto Rican”[tiab] OR ”Hispanic Cuban”[tiab] OR “non-English speaking”[tiab]) AND ("exclusion"[tiab] OR "inclusion"[tiab] OR "human subjects"[tiab] OR "participation in research"[tiab] OR "patient selection"[mesh] OR "recruit"[tiab] OR "recruited"[tiab] OR "recruiting"[tiab] OR "recruitment"[tiab] OR "research participant"[tiab] OR "research participants"[tiab] OR "research participation"[tiab] OR "research subjects"[mesh] OR "research subject"[tiab] OR "research subjects"[tiab] OR "underenroll"[tiab] OR "underenrolled"[tiab] OR "underrepresented"[tiab] OR under-representation[tiab] OR underrepresentation[tiab] OR under-enrollment[tiab] OR representation[tiab]) AND ( "2016/01/01"[PDat] : "2025/01/01"[PDat] ) AND English[la]) NOT (Animals[Mesh:noexp] OR review[ptyp] OR systematic[ti]) | 7500 |
|  | (("Cultural Diversity"[MeSH] OR "Ethnic Groups"[MeSH] OR "Minority Groups"[MeSH] OR "Minority Health"[MeSH] OR “Cultural diversity”[tiab] OR “Racial and ethnic minorities”[tiab] OR “Ethnic minorities”[tiab] OR “Racial minority”[tiab] OR “Racial differences”[tiab] OR “Racial differences”[tiab] OR “Racial”[tiab] OR “Ethnic minority”[tiab] OR “Ethnically diverse”[tiab] OR “Minorities”[tiab] OR “Underserved”[tiab] OR “Minority”[tiab] OR “Racially diverse”[tiab] OR “Diverse population”[tiab] OR "African Americans"[mesh] OR "african continental ancestry group"[mesh] OR “blacks”[tiab] OR “black”[tiab] OR “African-American”[tiab] OR "African Americans”[tiab] OR “ negro”[tiab] OR “negroes”[tiab] OR "negroid race”[tiab] OR “African ancestry”[tiab] OR “Hispanic Americans”[mesh] OR “Mexican Americans”[mesh] OR “Hispanic”[tiab] OR “Latino”[tiab] OR “Hispanics”[tiab] OR “Latinas”[tiab] OR “Latinos”[tiab] OR “Latina”[tiab] OR “Latino”[tiab] OR “Latin American”[tiab] OR “Mexican Americans”[tiab] OR ”Mexican American”[tiab] OR “chicanos”[tiab] OR “chicanas”[tiab] OR “chicano”[tiab] OR “chicana”[tiab] OR “Cuban descent”[tiab] OR “Puerto Rican descent”[tiab] OR “Mexican descent”[tiab] OR “Hispanic Puerto Rican”[tiab] OR ”Hispanic Cuban”[tiab] OR “non-English speaking”[tiab]) AND ("exclusion"[tiab] OR "inclusion"[tiab] OR "human subjects"[tiab] OR "participation in research"[tiab] OR "patient selection"[mesh] OR "recruit"[tiab] OR "recruited"[tiab] OR "recruiting"[tiab] OR "recruitment"[tiab] OR "research participant"[tiab] OR "research participants"[tiab] OR "research participation"[tiab] OR "research subjects"[mesh] OR "research subject"[tiab] OR "research subjects"[tiab] OR "underenroll"[tiab] OR "underenrolled"[tiab] OR "underrepresented"[tiab] OR under-representation[tiab] OR underrepresentation[tiab] OR under-enrollment[tiab] OR representation[tiab]) AND ( "2012/01/01"[PDat] : "2025/01/01"[PDat] ) AND English[la] AND (review[ptyp] OR systematic[tiab] OR review[tiab])) NOT Animals[Mesh:noexp] | 2663 |
| Sex | ((women[ti] OR woman[ti] OR female[ti] OR females[ti] OR man[ti] OR men[ti] OR male[ti] OR males[ti] OR sex[ti] OR gender[ti]) AND ("exclusion"[ti] OR "inclusion"[ti] OR "participation in research"[tiab] OR "recruit"[ti] OR "recruited"[tiab] OR "recruiting"[tiab] OR "recruitment"[tiab] OR "research participant"[tiab] OR "research participants"[tiab] OR "research participation"[tiab] OR "research subject"[tiab] OR "research subjects"[tiab] OR "underenroll"[tiab] OR "underenrolled"[tiab] OR "underrepresented"[tiab] OR under-representation[tiab] OR underrepresentation[tiab] OR under-enrollment[tiab]) AND ( "2012/01/01"[PDat] : "2025/01/01"[PDat] ) AND English[la] AND (review[ptyp] OR systematic[tiab] OR review[tiab])) NOT Animals[Mesh:noexp] | 212 |
|  | ("Sex"[Mesh] OR "Women"[Mesh] OR "Men"[Mesh] OR "women"[tiab] OR "men"[tiab]) AND ("research participation"[tiab] OR "under-representation"[tiab] OR “underrepresentation”[tiab] OR “underrepresented”[tiab] OR “under-represented”[tiab] OR “enrollment”[ti] OR “under-enrollment”[tiab] OR “diversity”[ti]) AND ("research participation"[tiab] OR "research recruitment"[tiab] OR "retention strategy"[tiab] OR ("participation in"[tiab] AND "research"[tiab])) AND (("2010/01/01"[PDAT] : "2050/12/31"[PDAT]) AND “English”[lang]) | 126 |
|  | ("Sex"[MeSH Terms] OR "sex"[tiab] OR "gender"[tiab]) AND ("research participation"[tiab] OR "under-representation"[tiab] OR "underrepresentation"[tiab] OR "underrepresented"[tiab] OR "under-represented"[tiab] OR "enrollment"[ti] OR "under-enrollment"[tiab] OR "diversity"[ti]) AND ("research participation"[tiab] OR "research recruitment"[tiab] OR "retention strategy"[tiab] OR ("participation in"[tiab] AND "research"[tiab])) AND (("2010/01/01"[PDAT] : "2050/12/31"[PDAT]) AND "English"[lang]) | 83 |
| Sexual Orientation | (Bisexuality[Mesh] OR "Homosexuality"[Mesh] OR homosexual[tiab] OR homosexuals[tiab] OR "Sexual Minorities"[Mesh] OR "sexual minority"[tiab] OR "sexual minorities"[tiab] OR "Homosexuality, Female"[Mesh] OR "Homosexuality, Male"[Mesh] OR lesbian[tiab] OR lesbians[tiab] OR gay[tiab] OR gays[tiab] bisexual[tiab] OR bisexuals[tiab] OR queer[tiab] OR queers[tiab] OR intersex[tiab] OR lgbt[tiab] OR lgbtq[tiab] OR "men who have sex with men"[tiab] OR "women who have sex with women"[tiab] OR wsw[tiab] OR msm[tiab] OR "sexual orientation"[tiab]) AND ("exclusion"[tiab] OR "inclusion"[tiab] OR "human subjects"[tiab] OR "participation in research"[tiab] OR "patient selection"[mesh] OR "recruit"[tiab] OR "recruited"[tiab] OR "recruiting"[tiab] OR "recruitment"[tiab] OR "research participant"[tiab] OR "research participants"[tiab] OR "research participation"[tiab] OR "research subjects"[mesh] OR "research subject"[tiab] OR "research subjects"[tiab] OR "underenroll"[tiab] OR "underenrolled"[tiab] OR "underrepresented"[tiab] OR under-representation[tiab] OR underrepresentation[tiab] OR under-enrollment[tiab] OR representation[tiab]) | 3514 |
