# Supplementary material for: Diversity and inclusion for the All of Us research program: A scoping review
Source: PLoS One. 2020 Jul 1;15(7):e0234962. doi: 10.1371/journal.pone.0234962 (PMC7329113; doi:10.1371/journal.pone.0234962)
Supplement: S2 Table — (DOCX) [file pone.0234962.s002.docx]

**S2 Table. Number of Articles Screened and Selected in Original Literature Search**

|  | **Reference Literature** | |
| --- | --- | --- |
| **Search Category** | **# Coded References** | **# of Final Selected** |
| Access to Care | 76 | 16 |
| Age | 55 | 28 |
| Annual Household Income | 65 | 13 |
| Disability | 49 | 16 |
| Education Attainment | 38 | 11 |
| Gender Identity* | 79 | 9 |
| Geography | 46 | 24 |
| Race and Ethnicity | 118 | 38 |
| Sex | 40 | 12 |
| Sexual Orientation* | 79 | 11 |
| *An additional 10 articles were selected that covered topics across the broader Sexual and Gender Minority domain.  These are included in the Appendix Bibliography as an additional subcomponent. | | |
