# Supplementary material for: Diversity and inclusion for the All of Us research program: A scoping review
Source: PLoS One. 2020 Jul 1;15(7):e0234962. doi: 10.1371/journal.pone.0234962 (PMC7329113; doi:10.1371/journal.pone.0234962)
Supplement: S1 File — (DOCX) [file pone.0234962.s003.docx]

**S1 File. Bibliography of Selected References by Search Category**

**Access to Care**

1. Langford M, Higgs G, Fry R. Multi-modal two-step floating catchment area analysis of primary health care accessibility. Health Place. 2016; 38:70-81.
2. Hudson BF, Flemming K, Shulman C, Candy B. Challenges to access and provision of palliative care for people who are homeless: a systematic review of qualitative research. BMC Palliat Care. 2016;15(1):96.
3. Campbell DJ, Manns BJ, Hemmelgarn BR, Sanmartin C, King-Shier KM. Development of a conceptual framework for understanding financial barriers to care among patients with cardiovascular-related chronic disease: a protocol for a qualitative (grounded theory) study. CMAJ Open. 2016;4(2): E304-8.
4. Neutens T. Accessibility, equity and health care: review and research directions for transport geographers. Journal of Transport Geography. 2015; 43:14-27.
5. Holden CD, Chen J, Dagher RK. Preventive care utilization among the uninsured by race/ethnicity and income. American journal of preventive medicine. 2015;48(1):13-21.
6. Egleston BL, Pedraza O, Wong YN, Dunbrack RL, Jr., Griffin CL, Ross EA, et al. Characteristics of clinical trials that require participants to be fluent in English. Clin Trials. 2015;12(6):618-26.
7. Tomsik PE, Smith S, Mason MJ, Zyzanski SJ, Stange KC, Werner JJ, et al. Understanding and measuring health care insecurity. J Health Care Poor Underserved. 2014;25(4):1821-32.
8. McGrail MR, Humphreys JS. Measuring spatial accessibility to primary health care services: Utilising dynamic catchment sizes. Applied Geography. 2014; 54:182-8.
9. Mao L, Nekorchuk D. Measuring spatial accessibility to healthcare for populations with multiple transportation modes. Health Place. 2013; 24:115-22.
10. Comber AJ, Brunsdon C, Radburn R. A spatial analysis of variations in health access: linking geography, socio-economic status and access perceptions. Int J Health Geogr. 2011; 10:44.
11. Carrillo JE, Carrillo VA, Perez HR, Salas-Lopez D, Natale-Pereira A, Byron AT. Defining and targeting health care access barriers. J Health Care Poor Underserved. 2011;22(2):562-75.
12. Kang-Kim M, Betancourt JR, Ayanian JZ, Zaslavsky AM, Yucel RM, Weissman JS. Access to care and use of preventive services by Hispanics: state-based variations from 1991 to 2004. Med Care. 2008;46(5):507-15.
13. Dhruva SS, Redberg RF. Variations between clinical trial participants and Medicare beneficiaries in evidence used for Medicare national coverage decisions. Arch Intern Med. 2008;168(2):136-40.
14. Probst JC, Laditka SB, Wang JY, Johnson AO. Effects of residence and race on burden of travel for care: cross sectional analysis of the 2001 US National Household Travel Survey. BMC Health Serv Res. 2007; 7:40.
15. Sherman JE, Spencer J, Preisser JS, Gesler WM, Arcury TA. A suite of methods for representing activity space in a healthcare accessibility study. Int J Health Geogr. 2005; 4:24.
16. Parker EB, Campbell JL. Measuring access to primary medical care: some examples of the use of geographical information systems. Health Place. 1998;4(2):183-93.

**Age**

1. Bayer A, Tadd W. Unjustified exclusion of elderly people from studies submitted to research ethics committee for approval: descriptive study. BMJ. 2000;321(7267):992-3.
2. Cherubini A, Del Signore S, Ouslander J, Semla T, Michel JP. Fighting against age discrimination in clinical trials. J Am Geriatr Soc. 2010;58(9):1791-6.
3. Cherubini A, Oristrell J, Pla X, Ruggiero C, Ferretti R, Diestre G, et al. The persistent exclusion of older patients from ongoing clinical trials regarding heart failure. Arch Intern Med. 2011;171(6):550-6.
4. Clegg A, Relton C, Young J, Witham M. Improving recruitment of older people to clinical trials: use of the cohort multiple randomised controlled trial design. Age and ageing. 2015;44(4):547-50.
5. Community Preventive Services Task F. Recommendation for Center-Based Early Childhood Education to Promote Health Equity. J Public Health Manag Pract. 2016;22(5):E9-E10.
6. Crome P, Lally F, Cherubini A, Oristrell J, Beswick AD, Clarfield AM, et al. Exclusion of older people from clinical trials: professional views from nine European countries participating in the PREDICT study. Drugs Aging. 2011;28(8):667-77.
7. Cruz-Jentoft AJ, Gutierrez B. Upper age limits in studies submitted to a research ethics committee. Aging Clin Exp Res. 2010;22(2):175-8.
8. Duncan RE, Drew SE, Hodgson J, Sawyer SM. Is my mum going to hear this? Methodological and ethical challenges in qualitative health research with young people. Soc Sci Med. 2009;69(11):1691-9.
9. Fitzsimmons PR, Blayney S, Mina-Corkill S, Scott GO. Older participants are frequently excluded from Parkinson's disease research. Parkinsonism Relat Disord. 2012;18(5):585-9.
10. Hoffman LF, Francis NK, Catallozzi M, Francis JK, Stanberry LR, Rosenthal SL. Inclusion of Adolescents in Clinical Trials for Sexually Transmitted Infections: A Review of Existing Registered Studies. The Journal of adolescent health: official publication of the Society for Adolescent Medicine. 2016;58(5):576-8.
11. Hutchins LF, Unger JM, Crowley JJ, Coltman CA, Jr., Albain KS. Underrepresentation of patients 65 years of age or older in cancer-treatment trials. N Engl J Med. 1999;341(27):2061-7.
12. Knechel NA. The challenges of enrolling older adults into intervention studies. The Yale journal of biology and medicine. 2013;86(1):41-7.
13. Konrat C, Boutron I, Trinquart L, Auleley GR, Ricordeau P, Ravaud P. Underrepresentation of elderly people in randomised controlled trials. The example of trials of 4 widely prescribed drugs. PLoS One. 2012;7(3): e33559.
14. Krumholz HM, Gross CP, Peterson ED, Barron HV, Radford MJ, Parsons LS, et al. Is there evidence of implicit exclusion criteria for elderly subjects in randomized trials? Evidence from the GUSTO-1 study. American Heart Journal. 2003;146(5):839-47.
15. Lewis JH, Kilgore ML, Goldman DP, Trimble EL, Kaplan R, Montello MJ, et al. Participation of patients 65 years of age or older in cancer clinical trials. J Clin Oncol. 2003;21(7):1383-9.
16. Mangione-Smith R, McGlynn EA. Assessing the quality of healthcare provided to children. Health services research. 1998;33(4 Pt 2):1059-90.
17. Masoudi FA, Havranek EP, Wolfe P, Gross CP, Rathore SS, Steiner JF, et al. Most hospitalized older persons do not meet the enrollment criteria for clinical trials in heart failure. American Heart Journal. 2003;146(2):250-7.
18. McMurdo ME, Witham MD, Gillespie ND. Including older people in clinical research. BMJ. 2005;331(7524):1036-7.
19. Provencher V, Mortenson WB, Tanguay-Garneau L, Belanger K, Dagenais M. Challenges and strategies pertaining to recruitment and retention of frail elderly in research studies: a systematic review. Archives of gerontology and geriatrics. 2014;59(1):18-24.
20. Rathore SS, Mehta RH, Wang Y, Radford MJ, Krumholz HM. Effects of age on the quality of care provided to older patients with acute myocardial infarction. Am J Med. 2003;114(4):307-15.
21. Rich MW, Chyun DA, Skolnick AH, Alexander KP, Forman DE, Kitzman DW, et al. Knowledge Gaps in Cardiovascular Care of the Older Adult Population: A Scientific Statement From the American Heart Association, American College of Cardiology, and American Geriatrics Society. Journal of the American College of Cardiology. 2016;67(20):2419-40.
22. Sanford SD, Beaumont JL, Snyder MA, Reichek J, Salsman JM. Clinical research participation among adolescent and young adults at an NCI-designated Comprehensive Cancer Center and affiliated pediatric hospital. Supportive care in cancer: official journal of the Multinational Association of Supportive Care in Cancer. 2017;25(5):1579-86.
23. Schmucker DL, Vesell ES. Are the elderly underrepresented in clinical drug trials? J Clin Pharmacol. 1999;39(11):1103-8.
24. Townsley CA, Selby R, Siu LL. Systematic review of barriers to the recruitment of older patients with cancer onto clinical trials. J Clin Oncol. 2005;23(13):3112-24.
25. Trimble EL, Carter CL, Cain D, Freidlin B, Ungerleider RS, Friedman MA. Representation of older patients in cancer treatment trials. Cancer. 1994;74(7 Suppl):2208-14.
26. Watts G. Why the exclusion of older people from clinical research must stop. BMJ. 2012;344: e3445.
27. Weintraub JA, Breland CE. Challenges, benefits, and factors to enhance recruitment and inclusion of children in pediatric dental research. International journal of paediatric dentistry. 2015;25(5):310-6.
28. Zulman DM, Sussman JB, Chen X, Cigolle CT, Blaum CS, Hayward RA. Examining the evidence: a systematic review of the inclusion and analysis of older adults in randomized controlled trials. J Gen Intern Med. 2011;26(7):783-90.

**Annual Household Income**

1. Adderley-Kelly B, Green PM. Strategies for successful conduct of research with low-income African American populations. Nurs Outlook. 2005;53(3):147-52.
2. Alsabbagh MH, Lemstra M, Eurich D, Lix LM, Wilson TW, Watson E, et al. Socioeconomic status and nonadherence to antihypertensive drugs: a systematic review and meta-analysis. Value Health. 2014;17(2):288-96.
3. Bonevski B, Randell M, Paul C, Chapman K, Twyman L, Bryant J, et al. Reaching the hard-to-reach: a systematic review of strategies for improving health and medical research with socially disadvantaged groups. BMC medical research methodology. 2014; 14:42.
4. Courtney RJ, Naicker S, Shakeshaft A, Clare P, Martire KA, Mattick RP. Smoking Cessation among Low-Socioeconomic Status and Disadvantaged Population Groups: A Systematic Review of Research Output. Int J Environ Res Public Health. 2015;12(6):6403-22.
5. Cui Z, Seburg EM, Sherwood NE, Faith MS, Ward DS. Recruitment and retention in obesity prevention and treatment trials targeting minority or low-income children: a review of the clinical trials registration database. Trials. 2015; 16:564.
6. Denny CC, Grady C. Clinical research with economically disadvantaged populations. J Med Ethics. 2007;33(7):382-5.
7. O'Mara-Eves A, Brunton G, Oliver S, Kavanagh J, Jamal F, Thomas J. The effectiveness of community engagement in public health interventions for disadvantaged groups: a meta-analysis. BMC Public Health. 2015; 15:129.
8. Pickett KE, Wilkinson RG. Income inequality and health: a causal review. Soc Sci Med. 2015; 128:316-26.
9. Sokol R, Fisher E, Hill J. Identifying Those Whom Health Promotion Hardly Reaches: A Systematic Review. Eval Health Prof. 2015;38(4):518-37.
10. Spencer N, Thanh TM, Louise S. Low income/socio-economic status in early childhood and physical health in later childhood/adolescence: a systematic review. Matern Child Health J. 2013;17(3):424-31.
11. Stone TH. The invisible vulnerable: the economically and educationally disadvantaged subjects of clinical research. The Journal of law, medicine & ethics: a journal of the American Society of Law, Medicine & Ethics. 2003;31(1):149-53.
12. UyBico SJ, Pavel S, Gross CP. Recruiting vulnerable populations into research: a systematic review of recruitment interventions. J Gen Intern Med. 2007;22(6):852-63.
13. Xu KT. State-level variations in income-related inequality in health and health achievement in the US. Soc Sci Med. 2006;63(2):457-64.

**Disability**

1. Bigby C, Frawley P, Ramcharan P. Conceptualizing inclusive research with people with intellectual disability. J Appl Res Intellect Disabil. 2014;27(1):3-12.
2. Brault MW. Americans with Disabilities: 2010. U.S Census Bureau; 2012.
3. Brooker K, van Dooren K, Tseng CH, McPherson L, Lennox N, Ware R. Out of sight, out of mind? The inclusion and identification of people with intellectual disability in public health research. Perspect Public Health. 2015;135(4):204-11.
4. Carlson L. Research ethics and intellectual disability: broadening the debates. The Yale journal of biology and medicine. 2013;86(3):303-14.
5. Chan L, Heinemann AW, Roberts J. Elevating the quality of disability and rehabilitation research: mandatory use of the reporting guidelines. Am J Occup Ther. 2014;68(2):127-9.
6. Coons K, Watson S. Conducting research with individuals who have intellectual disabilities: Ethical and practical implications for qualitative research2013. 14-24 p.
7. Corby D, Taggart L, Cousins W. People with intellectual disability and human science research: A systematic review of phenomenological studies using interviews for data collection. Res Dev Disabil. 2015; 47:451-65.
8. Feldman MA, Bosett J, Collet C, Burnham-Riosa P. Where are persons with intellectual disabilities in medical research? A survey of published clinical trials. J Intellect Disabil Res. 2014;58(9):800-9.
9. Harris J, Roberts K. Challenging Barriers to Participation in Qualitative Research: Involving Disabled Refugees. International Journal of Qualitative Methods. 2016;2(2):14-22.
10. Iacono T, Carling-Jenkins R. The human rights context for ethical requirements for involving people with intellectual disability in medical research. J Intellect Disabil Res. 2012;56(11):1122-32.
11. Jurkowski JM. Photovoice as participatory action research tool for engaging people with intellectual disabilities in research and program development. Intellectual and developmental disabilities. 2008;46(1):1-11.
12. Kembhavi G, Wirz S. Engaging adolescents with disabilities in research. Alter-European Journal of Disability Research. 2009;3(3):286-96.
13. Lamontagne ME, Perreault K, Gagnon MP. Evaluation of the acceptability, feasibility and effectiveness of two methods of involving patients with disability in developing clinical guidelines: study protocol of a randomized pragmatic pilot trial. Trials. 2014; 15:118.
14. Morrisey B. Ethics and research among persons with disabilities in long-term care. Qualitative health research. 2012;22(9):1284-97.
15. Taua C, Neville C, Hepworth J. Research participation by people with intellectual disability and mental health issues: an examination of the processes of consent. International journal of mental health nursing. 2014;23(6):513-24.
16. Williams AS, Moore SM. Universal design of research: inclusion of persons with disabilities in mainstream biomedical studies. Sci Transl Med. 2011;3(82):82cm12.

**Educational Attainment**

1. Byhoff E, Hamati MC, Power R, Burgard SA, Chopra V. Increasing educational attainment and mortality reduction: a systematic review and taxonomy. BMC Public Health. 2017;17(1):719.
2. Chiswick BR, DebBurman N. Educational attainment: analysis by immigrant generation. Economics of Education Review. 2004;23(4):361-79.
3. Cleveland RJ, Schwartz TA, Prizer LP, Randolph R, Schoster B, Renner JB, et al. Associations of educational attainment, occupation, and community poverty with hip osteoarthritis. Arthritis care & research. 2013;65(6):954-61.
4. Cohen AK, Rai M, Rehkopf DH, Abrams B. Educational attainment and obesity: a systematic review. Obesity reviews: an official journal of the International Association for the Study of Obesity. 2013;14(12):989-1005.
5. Demmler JC, Hill RA, Rahman MA, Bandyopadhyay A, Healy MA, Paranjothy S, et al. Educational Attainment at Age 10-11 Years Predicts Health Risk Behaviors and Injury Risk During Adolescence. The Journal of adolescent health: official publication of the Society for Adolescent Medicine. 2017;61(2):212-8.
6. Green JA, Cavanaugh KL. Understanding the influence of educational attainment on kidney health and opportunities for improved care. Advances in chronic kidney disease. 2015;22(1):24-30.
7. Huynh K. Risk factors: Low educational attainment linked to high CVD risk. Nat Rev Cardiol. 2017;14(8):442.
8. Johnson KE, Morris M, Rew L, Simonton AJ. A Systematic Review of Consent Procedures, Participation Rates, and Main Findings of Health-Related Research in Alternative High Schools From 2010 to 2015. The Journal of school nursing: the official publication of the National Association of School Nurses. 2016;32(1):20-31.
9. Kubota Y, Heiss G, MacLehose RF, Roetker NS, Folsom AR. Association of Educational Attainment With Lifetime Risk of Cardiovascular Disease: The Atherosclerosis Risk in Communities Study. JAMA internal medicine. 2017;177(8):1165-72.
10. Mikk J, Taht K, Must O. Sex differences in educational attainment. Personality and Individual Differences. 2012;53(2):132-6.
11. Montez JK, Zajacova A, Hayward MD. Disparities in Disability by Educational Attainment Across US States. Am J Public Health. 2017;107(7):1101-8.

**Gender Identity**

1. Carabez R, Pellegrini M, Mankovitz A, Eliason M, Scott M. Does your organization use gender inclusive forms? Nurses' confusion about trans* terminology. J Clin Nurs. 2015;24(21-22):3306-17.
2. Deutsch MB, Buchholz D. Electronic health records and transgender patients--practical recommendations for the collection of gender identity data. J Gen Intern Med. 2015;30(6):843-7.
3. Deutsch MB, Keatley J, Sevelius J, Shade SB. Collection of gender identity data using electronic medical records: survey of current end-user practices. J Assoc Nurses AIDS Care. 2014;25(6):657-63.
4. Meyer-Bahlburg HF. Sex steroids and variants of gender identity. Endocrinol Metab Clin North Am. 2013;42(3):435-52.
5. Pega F, Veale JF. The case for the World Health Organization's Commission on Social Determinants of Health to address gender identity. Am J Public Health. 2015;105(3): e58-62.
6. Reisner SL, Conron KJ, Tardiff LA, Jarvi S, Gordon AR, Austin SB. Monitoring the health of transgender and other gender minority populations: validity of natal sex and gender identity survey items in a U.S. national cohort of young adults. BMC Public Health. 2014; 14:1224.
7. Reisner SL, Katz-Wise SL, Gordon AR, Corliss HL, Austin SB. Social Epidemiology of Depression and Anxiety by Gender Identity. The Journal of adolescent health: official publication of the Society for Adolescent Medicine. 2016;59(2):203-8.
8. Schneider C, Cerwenka S, Nieder TO, Briken P, Cohen-Kettenis PT, De Cuypere G, et al. Measuring Gender Dysphoria: A Multicenter Examination and Comparison of the Utrecht Gender Dysphoria Scale and the Gender Identity/Gender Dysphoria Questionnaire for Adolescents and Adults. Arch Sex Behav. 2016;45(3):551-8.
9. Veale JF. Factorial Validity and Invariance Assessment of a Short Version of the Recalled Childhood Gender Identity/Role Questionnaire. Arch Sex Behav. 2016;45(3):537-50.

**Geography**

1. Reducing Health Disparities in Rural America: Key Provisions in the Affordable Care Act National Advisory Committee on Rural Health and Human Services; 2011.
2. Bennett KJ. Rural population estimates: an analysis of a large secondary data set. J Rural Health. 2013;29(3):233-8.
3. Bennett KJ, Pumkam C, Probst JC. Rural-urban differences in the location of influenza vaccine administration. Vaccine. 2011;29(35):5970-7.
4. Bergeron CD, Foster C, Friedman DB, Tanner A, Kim SH. Clinical trial recruitment in rural South Carolina: a comparison of investigators' perceptions and potential participant eligibility. Rural and remote health. 2013;13(4):2567.
5. Bigbee JL, Lind B. Methodological challenges in rural and frontier nursing research. Applied nursing research: ANR. 2007;20(2):104-6.
6. Blake KD, Moss JL, Gaysynsky A, Srinivasan S, Croyle RT. Making the Case for Investment in Rural Cancer Control: An Analysis of Rural Cancer Incidence, Mortality, and Funding Trends. Cancer Epidemiol Biomarkers Prev. 2017;26(7):992-7.
7. Bushy A. Conducting culturally competent rural nursing research. Annual review of nursing research. 2008; 26:221-36.
8. Cohen GI. Clinical research by community oncologists. CA Cancer J Clin. 2003;53(2):73-81.
9. Cohen SB, Braden JJ, Ward EP. Enhancing the representation of rural areas in the National Medical Expenditure Survey. J Rural Health. 1993;9(3):188-203.
10. Davis MM, Aromaa S, McGinnis PB, Ramsey K, Rollins N, Smith J, et al. Engaging the underserved: a process model to mobilize rural community health coalitions as partners in translational research. Clinical and translational science. 2014;7(4):300-6.
11. Dean C, Fogleman AJ, Zahnd WE, Lipka AE, Malhi RS, Delfino KR, et al. Engaging rural communities in genetic research: challenges and opportunities. J Community Genet. 2017;8(3):209-19.
12. Friedman DB, Bergeron CD, Foster C, Tanner A, Kim SH. What do people really know and think about clinical trials? A comparison of rural and urban communities in the South. Journal of community health. 2013;38(4):642-51.
13. Friedman DB, Foster C, Bergeron CD, Tanner A, Kim SH. A qualitative study of recruitment barriers, motivators, and community-based strategies for increasing clinical trials participation among rural and urban populations. Am J Health Promot. 2015;29(5):332-8.
14. Guadagnolo BA, Petereit DG, Helbig P, Koop D, Kussman P, Fox Dunn E, et al. Involving American Indians and medically underserved rural populations in cancer clinical trials. Clin Trials. 2009;6(6):610-7.
15. Harju BL, Wuensch KL, Kuhl EA, Cross NJ. Comparison of rural and urban residents' implicit and explicit attitudes related to seeking medical care. J Rural Health. 2006;22(4):359-63.
16. Hartley D. Rural health disparities, population health, and rural culture. Am J Public Health. 2004;94(10):1675-8.
17. Heaton LJ, Smith TA, Raybould TP. Factors influencing use of dental services in rural and urban communities: considerations for practitioners in underserved areas. Journal of dental education. 2004;68(10):1081-9.
18. Hohl SD, Gonzalez C, Carosso E, Ibarra G, Thompson B. "I did it for us and I would do it again": perspectives of rural latinos on providing biospecimens for research. Am J Public Health. 2014;104(5):911-6.
19. Kim SH, Tanner A, Friedman DB, Foster C, Bergeron CD. Barriers to clinical trial participation: a comparison of rural and urban communities in South Carolina. Journal of community health. 2014;39(3):562-71.
20. Mahnke AN, Plasek JM, Hoffman DG, Partridge NS, Foth WS, Waudby CJ, et al. A rural community's involvement in the design and usability testing of a computer-based informed consent process for the Personalized Medicine Research Project. Am J Med Genet A. 2014;164A(1):129-40.
21. Nieder C, Syed MA, Dalhaug A, Pawinski A, Norum J. Eligibility for phase 3 clinical trials of systemic therapy in real-world patients with metastatic renal cell cancer managed in a rural region. Med Oncol. 2017;34(9):149.
22. Pearce KA, Jarrett TD, Scutchfield FD, Talbert JC, Bolt WD, Barron MA, et al. Research Partnerships with Healthcare Providers in Rural Community Health Centers: Needs and Challenges in Diabetes Research. Public health frontier. 2015;4(1):1-6.
23. Virani S, Burke L, Remick SC, Abraham J. Barriers to recruitment of rural patients in cancer clinical trials. J Oncol Pract. 2011;7(3):172-7.
24. Young-Lorion J, Davis MM, Kirks N, Hsu A, Slater JK, Rollins N, et al. Rural Oregon community perspectives: introducing community-based participatory research into a community health coalition. Progress in community health partnerships: research, education, and action. 2013;7(3):313-22.

**Race and Ethnicity**

1. Advani AS, Atkeson B, Brown CL, Peterson BL, Fish L, Johnson JL, et al. Barriers to the participation of African-American patients with cancer in clinical trials: a pilot study. Cancer. 2003;97(6):1499-506.
2. Berger JS, Melloni C, Wang TY, Dolor RJ, Frazier CG, Samad Z, et al. Reporting and representation of race/ethnicity in published randomized trials. Am Heart J. 2009;158(5):742-7.
3. Corbie-Smith G, St George DM, Moody-Ayers S, Ransohoff DF. Adequacy of reporting race/ethnicity in clinical trials in areas of health disparities. J Clin Epidemiol. 2003;56(5):416-20.
4. Corbie-Smith G, Thomas SB, Williams MV, Moody-Ayers S. Attitudes and beliefs of African Americans toward participation in medical research. J Gen Intern Med. 1999;14(9):537-46.
5. Corbie-Smith G, Viscoli CM, Kernan WN, Brass LM, Sarrel P, Horwitz RI. Influence of race, clinical, and other socio-demographic features on trial participation. J Clin Epidemiol. 2003;56(4):304-9.
6. Djomand G, Katzman J, di Tommaso D, Hudgens MG, Counts GW, Koblin BA, et al. Enrollment of racial/ethnic minorities in NIAID-funded networks of HIV vaccine trials in the United States, 1988 to 2002. Public Health Rep. 2005;120(5):543-8.
7. Durant RW, Legedza AT, Marcantonio ER, Freeman MB, Landon BE. Different types of distrust in clinical research among whites and African Americans. J Natl Med Assoc. 2011;103(2):123-30.
8. Fisher JA, Kalbaugh CA. Challenging assumptions about minority participation in US clinical research. Am J Public Health. 2011;101(12):2217-22.
9. Ford JG, Howerton MW, Lai GY, Gary TL, Bolen S, Gibbons MC, et al. Barriers to recruiting underrepresented populations to cancer clinical trials: a systematic review. Cancer. 2008;112(2):228-42.
10. Garcia JA, Sanchez GR, Sanchez-Youngman S, Vargas ED, Ybarra VD. RACE AS LIVED EXPERIENCE: The Impact of Multi-Dimensional Measures of Race/Ethnicity on the Self-Reported Health Status of Latinos. Du Bois Rev. 2015;12(2):349-73.
11. Geller SE, Koch AR, Roesch P, Filut A, Hallgren E, Carnes M. The More Things Change, the More They Stay the Same: A Study to Evaluate Compliance With Inclusion and Assessment of Women and Minorities in Randomized Controlled Trials. Academic medicine: journal of the Association of American Medical Colleges. 2017.
12. George S, Duran N, Norris K. A systematic review of barriers and facilitators to minority research participation among African Americans, Latinos, Asian Americans, and Pacific Islanders. Am J Public Health. 2014;104(2): e16-31.
13. Ginther DK, Schaffer WT, Schnell J, Masimore B, Liu F, Haak LL, et al. Race, ethnicity, and NIH research awards. Science. 2011;333(6045):1015-9.
14. Grann VR. Erasing barriers to minority participation in cancer research. J Womens Health (Larchmt). 2010;19(5):837-8.
15. Grann VR, Jacobson JS, Troxel AB, Hershman D, Karp J, Myers C, et al. Barriers to minority participation in breast carcinoma prevention trials. Cancer. 2005;104(2):374-9.
16. Hussain-Gambles M, Atkin K, Leese B. Why ethnic minority groups are under-represented in clinical trials: a review of the literature. Health Soc Care Community. 2004;12(5):382-8.
17. Hussain-Gambles M, Leese B, Atkin K, Brown J, Mason S, Tovey P. Involving South Asian patients in clinical trials. Health Technol Assess. 2004;8(42): iii, 1-109.
18. James RD, Yu JH, Henrikson NB, Bowen DJ, Fullerton SM, Health Disparities Working G. Strategies and stakeholders: minority recruitment in cancer genetics research. Community Genet. 2008;11(4):241-9.
19. Kwiatkowski K, Coe K, Bailar JC, Swanson GM. Inclusion of minorities and women in cancer clinical trials, a decade later: Have we improved? Cancer. 2013;119(16):2956-63.
20. Lee C. "Race" and "ethnicity" in biomedical research: how do scientists construct and explain differences in health? Soc Sci Med. 2009;68(6):1183-90.
21. Lim E, Miyamura J, Chen JJ. Racial/Ethnic-Specific Reference Intervals for Common Laboratory Tests: A Comparison among Asians, Blacks, Hispanics, and White. Hawaii J Med Public Health. 2015;74(9):302-10.
22. Ma IW, Khan NA, Kang A, Zalunardo N, Palepu A. Systematic review identified suboptimal reporting and use of race/ethnicity in general medical journals. J Clin Epidemiol. 2007;60(6):572-8.
23. Mathew SS, Barwell J, Khan N, Lynch E, Parker M, Qureshi N. Inclusion of diverse populations in genomic research and health services: Genomix workshop report. J Community Genet. 2017;8(4):267-73.
24. Oh SS, Galanter J, Thakur N, Pino-Yanes M, Barcelo NE, White MJ, et al. Diversity in Clinical and Biomedical Research: A Promise Yet to Be Fulfilled. PLoS Med. 2015;12(12):e1001918.
25. Park SS, Grayson MH. Clinical research: protection of the "vulnerable"? J Allergy Clin Immunol. 2008;121(5):1103-7.
26. Popejoy AB, Fullerton SM. Genomics is failing on diversity. Nature. 2016;538(7624):161-4.
27. Roberson NL. Clinical trial participation. Viewpoints from racial/ethnic groups. Cancer. 1994;74(9 Suppl):2687-91.
28. Sengupta S, Lo B, Strauss RP, Eron J, Gifford AL. How researchers define vulnerable populations in HIV/AIDS clinical trials. AIDS Behav. 2010;14(6):1313-9.
29. Sengupta S, Strauss RP, DeVellis R, Quinn SC, DeVellis B, Ware WB. Factors affecting African-American participation in AIDS research. J Acquir Immune Defic Syndr. 2000;24(3):275-84.
30. Simon MA, de la Riva EE, Bergan R, Norbeck C, McKoy JM, Kulesza P, et al. Improving diversity in cancer research trials: the story of the Cancer Disparities Research Network. J Cancer Educ. 2014;29(2):366-74.
31. Somerson JS, Bhandari M, Vaughan CT, Smith CS, Zelle BA. Lack of diversity in orthopaedic trials conducted in the United States. J Bone Joint Surg Am. 2014;96(7): e56.
32. Spratt DE, Chan T, Waldron L, Speers C, Feng FY, Ogunwobi OO, et al. Racial/Ethnic Disparities in Genomic Sequencing. JAMA oncology. 2016;2(8):1070-4.
33. Taran FA, Brown HL, Stewart EA. Racial diversity in uterine leiomyoma clinical studies. Fertil Steril. 2010;94(4):1500-3.
34. Waheed W, Hughes-Morley A, Woodham A, Allen G, Bower P. Overcoming barriers to recruiting ethnic minorities to mental health research: a typology of recruitment strategies. BMC psychiatry. 2015;15(1):101.
35. Welch MJ, Lally R, Miller JE, Pittman S, Brodsky L, Caplan AL, et al. The ethics and regulatory landscape of including vulnerable populations in pragmatic clinical trials. Clin Trials. 2015;12(5):503-10.
36. Wendler D, Kington R, Madans J, Van Wye G, Christ-Schmidt H, Pratt LA, et al. Are racial and ethnic minorities less willing to participate in health research? PLoS Med. 2006;3(2): e19.
37. Yancey AK, Ortega AN, Kumanyika SK. Effective recruitment and retention of minority research participants. Annu Rev Public Health. 2006; 27:1-28.

**Sex**

1. Beery AK, Zucker I. Sex bias in neuroscience and biomedical research. Neurosci Biobehav Rev. 2011;35(3):565-72.
2. Ding EL, Powe NR, Manson JE, Sherber NS, Braunstein JB. Sex differences in perceived risks, distrust, and willingness to participate in clinical trials: a randomized study of cardiovascular prevention trials. Arch Intern Med. 2007;167(9):905-12.
3. Kim AM, Tingen CM, Woodruff TK. Sex bias in trials and treatment must end. Nature. 2010;465(7299):688-9.
4. Kragholm K, Halim SA, Yang Q, Schulte PJ, Hochman JS, Melloni C, et al. Sex-Stratified Trends in Enrollment, Patient Characteristics, Treatment, and Outcomes Among Non-ST-Segment Elevation Acute Coronary Syndrome Patients: Insights From Clinical Trials Over 17 Years. Circ Cardiovasc Qual Outcomes. 2015;8(4):357-67.
5. Mansukhani NA, Yoon DY, Teter KA, Stubbs VC, Helenowski IB, Woodruff TK, et al. Determining If Sex Bias Exists in Human Surgical Clinical Research. JAMA Surg. 2016;151(11):1022-30.
6. Nolan MR, Nguyen TL. Analysis and reporting of sex differences in phase III medical device clinical trials-how are we doing? J Womens Health (Larchmt). 2013;22(5):399-401.
7. Pilote L, Dasgupta K, Guru V, Humphries KH, McGrath J, Norris C, et al. A comprehensive view of sex-specific issues related to cardiovascular disease. CMAJ. 2007;176(6): S1-44.
8. Powers MS, Smith PH, McKee SA, Ehringer MA. From sexless to sexy: Why it is time for human genetics to consider and report analyses of sex. Biol Sex Differ. 2017; 8:15.
9. Sandberg K, Verbalis JG. Sex and the basic scientist: is it time to embrace Title IX? Biol Sex Differ. 2013;4(1):13.
10. Segarra I, Modamio P, Fernandez C, Marino EL. Sex-Divergent Clinical Outcomes and Precision Medicine: An Important New Role for Institutional Review Boards and Research Ethics Committees. Front Pharmacol. 2017; 8:488.
11. Tannenbaum C, Schwarz JM, Clayton JA, de Vries GJ, Sullivan C. Evaluating sex as a biological variable in preclinical research: the devil in the details. Biol Sex Differ. 2016; 7:13.
12. Weinberger AH, Smith PH, Kaufman M, McKee SA. Consideration of sex in clinical trials of transdermal nicotine patch: a systematic review. Exp Clin Psychopharmacol. 2014;22(5):373-83.

**Sexual Orientation**

1. Sexual Orientation and the 2011 Census UK Background Information. 2006.
2. Auer MK, Fuss J, Hohne N, Stalla GK, Sievers C. Transgender transitioning and change of self-reported sexual orientation. PLoS One. 2014;9(10): e110016.
3. Black D, Gates G, Sanders S, Taylor L. Demographics of the gay and lesbian population in the United States: evidence from available systematic data sources. Demography. 2000;37(2):139-54.
4. Byles JE, Forder PM, Grulich A, Prestage G. "It's okay to ask." Inclusion of sexual orientation questions is feasible in population health surveys. Aust N Z J Public Health. 2013;37(4):390-1.
5. Egleston BL, Dunbrack RL, Jr., Hall MJ. Clinical trials that explicitly exclude gay and lesbian patients. N Engl J Med. 2010;362(11):1054-5.
6. Fredriksen-Goldsen KI, Kim HJ. Count me in: response to sexual orientation measures among older adults. Res Aging. 2015;37(5):464-80.
7. Mattocks KM, Sullivan JC, Bertrand C, Kinney RL, Sherman MD, Gustason C. Perceived Stigma, Discrimination, and Disclosure of Sexual Orientation Among a Sample of Lesbian Veterans Receiving Care in the Department of Veterans Affairs. LGBT Health. 2015;2(2):147-53.
8. Mitchell M, Howarth C, Kotecha M, Kreegan C. Sexual orientation research review 2008. Equality and Human Rights Commission; 2008.
9. Ott MQ, Corliss HL, Wypij D, Rosario M, Austin SB. Stability and change in self-reported sexual orientation identity in young people: application of mobility metrics. Arch Sex Behav. 2011;40(3):519-32.
10. Pega F, Gray A, Veale JF, Binson D, Sell RL. Toward global comparability of sexual orientation data in official statistics: a conceptual framework of sexual orientation for health data collection in New Zealand's official statistics system. J Environ Public Health. 2013; 2013:473451.
11. Sell RL, Becker JB. Sexual orientation data collection and progress toward Healthy People 2010. Am J Public Health. 2001;91(6):876-82.

***Additional articles that cover topics across the Sexual and Gender Minority Domain***

1. The Health of Lesbian, Gay, Bisexual, and Transgender People: Building a Foundation for Better Understanding. Committee on Lesbian G, Bisexual, and Transgender Health Issues and Research Gaps and Opportunities; Board on the Health of Select Populations; Institute of Medicine, editor. Washington (DC)2011.
2. Boehmer U. Twenty years of public health research: inclusion of lesbian, gay, bisexual, and transgender populations. Am J Public Health. 2002;92(7):1125-30.
3. Coulter RW, Kenst KS, Bowen DJ, Scout. Research funded by the National Institutes of Health on the health of lesbian, gay, bisexual, and transgender populations. Am J Public Health. 2014;104(2): e105-12.
4. Eckstrand KL, Lunn MR, Yehia BR. Applying Organizational Change to Promote Lesbian, Gay, Bisexual, and Transgender Inclusion and Reduce Health Disparities. LGBT Health. 2017;4(3):174-80.
5. Fisher CB, Mustanski B. Reducing Health Disparities and Enhancing the Responsible Conduct of Research Involving LGBT Youth. The Hastings Center report. 2014;44 Suppl 4: S28-31.
6. Hughes RL, Damin C, Heiden-Rootes K. Where's the LGBT in integrated care research? A systematic review. Families, systems & health: the journal of collaborative family healthcare. 2017;35(3):308-19.
7. Institute of Medicine Committee on Lesbian GB, Transgender Health I, Research G, Opportunities. The National Academies Collection: Reports funded by National Institutes of Health. Washington (DC): National Academies Press (US), National Academy of Sciences.; 2011.
8. Lee JG, Ylioja T, Lackey M. Identifying Lesbian, Gay, Bisexual, and Transgender Search Terminology: A Systematic Review of Health Systematic Reviews. PLoS One. 2016;11(5): e0156210.
9. Patterson JG, Jabson JM, Bowen DJ. Measuring Sexual and Gender Minority Populations in Health Surveillance. LGBT Health. 2017;4(2):82-105.
10. Sell RL, Dunn PM. Inclusion of lesbian, gay, bisexual and transgender people in tobacco use-related surveillance and epidemiological research. J LGBT Health Res. 2008;4(1):27-42.

***Additional articles selected in updated search of the literature conducted in January 2020***

1. Dunn C, Wilson A, Sitas F. Older cancer patients in cancer clinical trials are underrepresented. Systematic literature review of almost 5000 meta- and pooled analyses of phase III randomized trials of survival from breast, prostate and lung cancer. Cancer Epidemiol. 2017;51:113–7.
2. Desmarais P, Miville C, Milán-Tomás Á, Nguyen QD, Ojeda-López C, Masellis M, et al. Age representation in antiepileptic drug trials: A systematic review and meta-analysis. Epilepsy Res. 2018;142:9–15.
3. Alwadi MA, Baker SR, Owens J. The inclusion of children with disabilities in oral health research: A systematic review. Community Dent Oral Epidemiol. 2018;46(3):238–44.
4. Ojha RP, Jackson BE, Lu Y, Burton M, Blair SE, MacDonald BR, et al. Participation and retention can be high in randomized controlled trials targeting underserved populations: a systematic review and meta-analysis. J Clin Epidemiol. 2018;98:154–7.
5. Macleod AD, Henery R, Nwajiugo PC, Scott NW, Caslake R, Counsell CE. Age-related selection bias in Parkinson’s disease research: are we recruiting the right participants? Parkinsonism Relat Disord. 2018;55:128–33.
6. Tahhan AS, Vaduganathan M, Greene SJ, Fonarow GC, Fiuzat M, Jessup M, et al. Enrollment of Older Patients, Women, and Racial and Ethnic Minorities in Contemporary Heart Failure Clinical Trials: A Systematic Review. JAMA Cardiol. 2018 01;3(10):1011–9.
7. Haughton CF, Silfee VJ, Wang ML, Lopez-Cepero AC, Estabrook DP, Frisard C, et al. Racial/ethnic representation in lifestyle weight loss intervention studies in the United States: A systematic review. Prev Med Rep. 2018 Mar;9:131
8. Gilmore-Bykovskyi AL, Jin Y, Gleason C, Flowers-Benton S, Block LM, Dilworth-Anderson P, et al. Recruitment and retention of underrepresented populations in Alzheimer’s disease research: A systematic review. Alzheimers Dement (N Y). 2019;5:751–70.
9. Unger JM, Vaidya R, Hershman DL, Minasian LM, Fleury ME. Systematic Review and Meta-Analysis of the Magnitude of Structural, Clinical, and Physician and Patient Barriers to Cancer Clinical Trial Participation. J Natl Cancer Inst. 2019 Mar 1;111(3):245–55.
10. Fisher ER, Pratt R, Esch R, Kocher M, Wilson K, Lee W, et al. The role of race and ethnicity in views toward and participation in genetic studies and precision medicine research in the United States: A systematic review of qualitative and quantitative studies. Mol Genet Genomic Med. 2019 Dec 23;e1099.
11. Siembida EJ, Loomans-Kropp HA, Trivedi N, O’Mara A, Sung L, Tami-Maury I, et al. Systematic review of barriers and facilitators to clinical trial enrollment among adolescents and young adults with cancer: Identifying opportunities for intervention. Cancer. 2019 Dec 23;
12. Morone J. Systematic review of sociodemographic representation and cultural responsiveness in psychosocial and behavioral interventions with adolescents with type 1 diabetes. J Diabetes. 2019 Jul;11(7):582-592.
