# Supplementary material for: Diversity and inclusion for the All of Us research program: A scoping review
Source: PLoS One. 2020 Jul 1;15(7):e0234962. doi: 10.1371/journal.pone.0234962 (PMC7329113; doi:10.1371/journal.pone.0234962)
Supplement: S2 File — (DOCX) [file pone.0234962.s004.docx]

**S2 File. Past and Present *All of Us* Research Program Principle Investigators**

Brian Ahmedani, PhD, MSW^1^; Christine D Cole Johnson, PhD, MPH^1^; Habib Ahsan, MD, MMedSc^2^; Donna Antoine-LaVigne, PhD, MPH, MSEd^3^; Glendora Singleton^3^; Hoda Anton-Culver, PhD^4^; Eric Topol, MD^5^; Katie Baca-Motes, MBA^5^; Steven Steinhubl, MD^5^; James Wade, MD^6^; Mark Begale^6^; Praduman Jain, MSEE^6^; Scott Sutherland^6^; Beth Lewis^7^; Bruce Korf, MD, PhD^7^; Melissa Behringer, MD*^7^; Ali G Gharavi, MD^8^; David B Goldstein, PhD^8^; George Hripcsak, MD, MS^8^; Louise Bier, MS^8^; Eric Boerwinkle, PhD, MS, MA^9^; Murray H Brilliant, PhD*^10^; Narayana Murali^10^; Scott Joseph Hebbring^10^; Dorothy Farrar-Edwards, PhD^11^; Elizabeth Burnside^11^; Marc K Drezner, MD*^11^; Amy Taylor^12^; Veena Channamsetty, MD*^12^; Wanda Montalvo, PhD, RN*^12^; Yashoda Sharma, PhD^12^; Carmen Chinea, MD, MPH^13^; Nancy Jenks, MS, CFNP, FAANP^13^; Mine Cicek, PhD^14^; Steve Thibodeau^14^; Beverly Wilson Holmes, MSW^15^; Eric Schlueter, MD^15^; Ever Collier, NP*^15^; Joyce Winkler, MPH*^15^; John Corcoran, MD^16^; Nick D’Addezio^17^; Martha Daviglus, MD, PhD^18^; Robert Winn, MD*^18^; Consuelo Wilkins, MD, MSCI^19^; Dan Roden, MD, CM^19^; Joshua Denny, MD, MS*^19^; Kim Doheny^20^; Debbie Nickerson, PhD^21^; Evan Eichler^21^; Gail Jarvik, MD, PhD^21^; Gretchen Funk^22^; Anthony Philippakis, MD, PhD^23^; Heidi Rehm, PhD, MMSc, FACMG^23^; Niall Lennon^23^; Sekar Kathiresan, MD*^23^; Stacey Gabriel, PhD^23^; Richard Gibbs^24^; Edgar M Gil Rico, MBA, MSc^25^; David Glazer^26^; Joannie Grand, MSN, RN*^27^; Philip Greenland, MD^28^; Paul Harris, PhD^+29^; Elizabeth Shenkman, PhD^30^; William R Hogan, MD, MS^30^; Priscilla Igho-Pemu, MD, MSCR, FACP^31^; Cliff Pollan^32^; Milena Jorge*^32^; Sally Okun, MMHS, RN*^32^; Elizabeth W Karlson, MD^33^; Jordan Smoller, MD, ScD^33^; Shawn N Murphy, MD, PhD^33^; Margaret Elizabeth Ross, MD, PhD^34^; Rainu Kaushal, MD, MPH^34^; Eboni Winford, PhD^35^; Febe Wallace, MD^35^; Parinda Khatri, PhD^35^; Vik Kheterpal^36^; Akinlolu Ojo, MD, PhD, MPH, MBA*^37^; Francisco A Moreno, MD^37^; Irving Kron^37^; Rachele Peterson, MS*^37^; Usha Menon, PhD, RN, FAAN*^37^; Patricia Watkins Lattimore^38^; Noga Leviner^39^; Juno Obedin-Maliver^40^; Mitchell Lunn, MD, MAS, FASN^40^; Lynda Malik-Gagnon^41^; Lara Mangravite, PhD^42^; Adria Marallo^43^; Oscar Marroquin, MD44; Shyam Visweswaran, MD, PhD44; Steven Reis, MD44; Gailen Marshall, Jr., MD, PhD*^45^; Patrick McGovern^46^; Deb Mignucci^47^; John Moore^48^; Fatima Munoz, MD, MPH^49^; Gregory Talavera, MD, MPH^49^; George T O'Connor, MD, MS^50^; Christopher O'Donnell, MD, MPH^51^; Lucila Ohno-Machado, MD, PhD^52^; Greg Orr^53^; Fornessa Randal, MCRP^54^; Andreas A Theodorou, MD^55^; Eric Reiman, MD^55^; Mercedita Roxas-Murray^56^; Louisa Stark^57^; Ronnie Tepp, MPP^58^; Alicia Zhou, PhD^59^; Scott Topper, PhD, FACMG^59^; Rhonda Trousdale, MD^60^; Phil Tsao, PhD^61^; Lisa Weidman^62^; Scott T Weiss, MD, MS^63^; David Wellis, PhD^64^; Jeffrey Whittle, MD, MPH^65^; Amanda Wilson, MS^66^; Stephan Zuchner, MD, PhD^67^; Michael E Zwick, PhD^68^

Legend

*Past Principal Investigator

+ Principal Investigator/Lead Author for the *All of Us* Research Program protocol ([paul.a.harris@vumc.org](mailto:paul.a.harris@vumc.org))

Affiliations

1. Henry Ford Health System

2. University of Chicago Medical Center

3. Jackson-Hinds Comprehensive Health Center

4. University of California, Irvine

5. Scripps Research Translational Institute

6. Vibrent Health

7. University of Alabama at Birmingham

8. Columbia University

9. University of Texas Health Science Center at Houston

10. Marshfield Clinic Research Institute

11. University of Wisconsin at Madison

12. Community Health Center, Inc.

14. Mayo Clinic and Foundation, Rochester

15. Cooperative Health

16. EMSI

17. Blue Cross Blue Shield Association

18. University of Illinois at Chicago

19. Vanderbilt University Medical Center

20. Johns Hopkins University School of Medicine

21. University of Washington

22. FiftyForward

23. Broad Institute

24. Baylor University

25. National Alliance for Hispanic Health

26. Verily Life Sciences

27. Mitre Corporation

28. Northwestern University

29. Vanderbilt University Medical Center

30. University of Florida

31. Morehouse School of Medicine, Atlanta

32. PatientsLikeMe

33. Partners Health Care

34. Cornell University, Weill Medical College

35. Cherokee Health Systems

36. CareEvolution, Inc.

37. University of Arizona, Tucson

38. Delta Research and Educational Foundation

39. PicnicHealth

40. Stanford University

41. DXC Technology

42. Sage Bionetworks

43. Quest Diagnostics Incorporated

44. University of Pittsburgh

45. University of Mississippi Medical Center

46. Wondros

47. WebMD Health Corporation

48. Fitbit, Inc.

49. San Ysidro Health Center

50. Boston Medical Center

51. VA AoU Coordinating Center - Boston

52. University of California, San Diego

53. Walgreen Co.

54. Asian Health Coalition

55. Banner Health

56. Montage Marketing Group

57. University of Utah

58. HCM Strategists

59. Color Genomics, Inc.

60. NYC Health + Hospitals

61. VA AoU Coordinating Center - Palo Alto

62. QTC

63. Brigham and Women's Hospital

64. San Diego Blood Bank

65. Medical College of Wisconsin

66. National Library of Medicine (NLM)

67. University of Miami

68. Emory University
