# Supplementary material for: Diversity and inclusion for the All of Us research program: A scoping review
Source: PLoS One. 2020 Jul 1;15(7):e0234962. doi: 10.1371/journal.pone.0234962 (PMC7329113; doi:10.1371/journal.pone.0234962)
Supplement: Attachment — Submitted filename: Response to Reviewers.docx [file pone.0234962.s005.docx]

Title: Diversity and Inclusion for the All of Us Research Program: A Scoping Review

Manuscript number: PONE-D-19-34035

**Reviewer #1:** I have only one strong recommendation for the authors. There is a brief mention of the role of lack of trust in the low participation rate of Black and African American participants in biomedical research. This is concerning, as there is a growing body of evidence (e.g., [https://doi.org/10.1002/cncr.28483](https://nam05.safelinks.protection.outlook.com/?url=https%3A%2F%2Fdoi.org%2F10.1002%2Fcncr.28483&data=02%7C01%7Cbrandy.mapes%40vumc.org%7C26eb9058e0c8462c426408d7e2535017%7Cef57503014244ed8b83c12c533d879ab%7C0%7C1%7C637226719776034413&sdata=uha3MIrCfugb98DfGgVAL1FK4YZ8JpQx%2Fz2%2Fp%2BJll1s%3D&reserved=0)) that when there is equal access to participate in and individuals are explicitly ASKED to participate in clinical trials, African Americans are no less likely to agree to participate nor to be willing to participate in clinical trials research. I fear that not including this in the current manuscript may irresponsibly perpetuate the lack-of-trust excuse that researchers often use to justify a lack of diversity in their research. We often characterize groups as “hard-to-reach” for any number of reasons, when in reality what we are saying is that they are just “easy to ignore.” I believe that the authors of this manuscript are working to change this norm in biomedical research, so I think that it is important to recognize how citing trust plays a role. Similarly, I think it is important to include any evidence we have for whether when other underrepresented groups – those from rural areas, those with disabilities, etc. -- are offered equal access to clinical trials they will accept, but this may be an important point to include in this paper to support the authors’ efforts to shift norms towards including UBR groups in research.

**Response:** Thank you for raising this point as it is important to highlight. We acknowledge as well that there is evidence supporting the argument that black and African American individuals are not less likely to participate in clinical trial research, particularly cancer trials, when explicitly asked. Clinical trials, however, are associated with better care and more potential for therapeutic benefit than other types of research activities. We believe there are two issues with trust to note – there is a lack of trust (participant) and not being trustworthy (researchers/institutions). Inviting individuals to participate, in a respectful way, may aid in overcoming the lack of trust barrier. This may not mean, however, that the barrier does not exist. We have addressed this point and clarified in the manuscript that the reason to cite low trust is so that researchers become more trustworthy (<https://www.ncbi.nlm.nih.gov/pmc/articles/PMC6143205/>) and that racial differences in trust are not just historically important to note (<https://www.ncbi.nlm.nih.gov/pmc/articles/PMC6802409/>).

**Reviewer #1:**  Can you provide an explanation for how “Individuals who have had a needed medical visit in the past 12 months” is representative of access to care? Does this mean that they went to a medical facility, therefore they have access to care? It was unclear to me how this represents a lack of access to care.

**Response:** Access to care can be measured according to several major elements including timeliness of receiving care when a need arises. This element is often measured according to one’s ability to receive care when needed over the course of a 12 month timespan.* This means the individual had the resources required to receive care when needed (ex. Transportation, money, locality to a medical care clinic, etc.) The *All of Us* Research Program recommends that both individuals who have not received a needed medical visit in the past 12 months or cannot easily obtain or pay for care as needed should be considered underrepresented in biomedical research.

*1. Elements of Access to Health Care | Agency for Health Research and Quality [Internet]. [cited 2020 Apr 19]. Available from: <https://www.ahrq.gov/research/findings/nhqrdr/chartbooks/access/elements.html>

**Reviewer #1:** In Figure 1, it is very difficult to distinguish the lines in the charts (and therefore the reported proportions of race and ethnicity) because of the colors. Also, why does the projected population of Native Hawaiian and Pacific Islanders reach 0%? Are they eliminating that category from the census?

**Response:** Figure 1 has been revised so that the lines are wider and easier to distinguish. Please note that the project population of Native Hawaiian and Pacific Islanders are as follows: 0.2% (2016), 0.2% (2020), 0.3% (2030), 0.3% (2040). The figure originally submitted listed the 2020 population as 4% in error.

**Reviewer #1:** Are there any actual study participants included in the Participant Provided Information Committee, particularly those representing any of the groups in the proposed guidelines? If so, this could certainly be a strength to include in the manuscript.

**Response:** Yes, more than 30 participant representatives are active members of the *All of Us* governance committees. The Participant Provided Information Committee roster includes two participant partners. This is now noted in the manuscript.

**Reviewer #1:** Minor comments:

1. All the figures and tables are difficult to read, even as the TIFF files.

2. Line 188 need space between “and” and “170”

3. Line 209, 220, 224, possibly other instances: Data are plural, so data “were” not “was”

**Response:** Thank you for this feedback.

1. The figures and tables have been revised to improve readability. Figure 3 has been repurposed as a table (Table 3).
2. This grammatical error has been corrected.
3. These corrections have also been made.
